# Axenfeld-Rieger syndrome: a novel histopathologic finding associated with corneal abnormalities

**DOI:** 10.1186/s12886-022-02754-8

**Published:** 2022-12-28

**Authors:** Ting Yu, Zhihao Dai, Rongmei Peng, Gege Xiao, Pei Zhang, Siyi Ma, Jing Hong

**Affiliations:** 1 Department of Ophthalmology, Peking University Third Hospital, No. 49 Garden North Road, Haidian 100191 Beijing, China; 2 Beijing Key Laboratory of Restoration of Damaged Ocular Nerve, Peking University Third Hospital, Beijing, China

**Keywords:** Axenfeld-Rieger syndrome, Histopathology, Congenital corneal opacity, Endothelial decompensation

## Abstract

**Background:**

Axenfeld-Rieger syndrome (ARS) is a rare kind of anterior segment dysgenesis (ASD). The most common ocular features of ARS are posterior embryotoxon and iris hypoplasia, while some patients may manifest as corneal opacity and edema. However, the current understanding of how ARS affects the cornea is still incomplete. This study reports a novel histopathological finding of ARS, complicating corneal abnormalities, including congenital corneal opacity and irreversible endothelial decompensation.

**Methods:**

This retrospective study included 6 eyes of 3 ARS patients, 5 of which underwent keratoplasty for irreversible endothelial decompensation from May 2016 to January 2019. No eye had a history of surgery. We reviewed the data of epidemiology, clinical manifestations and histopathologic examinations.

**Results:**

Five eyes developed irreversible endothelial decompensation, among which 4 were born with corneal opacity. One eye exhibited transparent cornea but showed a continuous loss of endothelial cells in the absence of surgery and elevated intraocular pressure thereafter. Anterior segment optical coherence tomography photographs showed that anterior synechia existed in the area with corneal opacities, where we found the interlayer splitting of the Descemet membrane inserted by hypoplastic iris and a basement membrane-like structure under a light microscope.

**Conclusion:**

Anterior synechia might be associated with corneal abnormalities in ARS patients. The novel histopathologic finding revealed the internal relation between anterior segment dysgenesis and would help explore the inner mechanism of corneal abnormalities in ARS.

## Introduction

Axenfeld-Rieger syndrome (ARS) is a rare kind of anterior segment dysgenesis (ASD) with an estimated prevalence of 1 in 50,000 to 100,000 newborn babies [1]. The main pathogenesis of ARS is abnormal migration and differentiation of neural crest cells (NCCs) during the embryonic period, leading to multiple phenotypes of ARS [2]. ARS is the general term for a contiguous clinical spectrum of developmental disorders. In the past, ARS was classified into four categories: Axenfeld anomaly (AA), Axenfeld syndrome (AS), Rieger anomaly (RA), and Rieger syndrome (RS). Patients with posterior embryotoxon (caused by anteriorly displaced Schwalbe’s line) and peripheral anterior adhesions were diagnosed as AA, while those with the addition of iris hypoplasia (especially corectopia and polycoria) were diagnosed as RA. Moreover, patients with accompanying systemic defects, including mild midface abnormalities, redundant umbilical skin, dental and cardiovascular abnormalities, were diagnosed as AS and RS respectively [3]. However, as the four categories have overlapping combinations of ocular and systemic abnormalities, and can result from mutations of the same genes, it is not necessary to have a separate term for ARS [1].

Corneal abnormalities, such as corneal opacity and edema, are considered the atypical features of ARS, which are caused by glaucoma or intraocular surgery in most reported cases [4–8]. Congenital corneal opacity (CCO) is rarely observed in ARS, while the exact mechanism remains unknown [9–11]. A study reported that CCO seen in ARS and other ASD (especially Peters anomaly, PA) could stem from a similar underlying mechanism [12]. Besides, some patients with unexplained corneal endothelial abnormalities have been reported, but all of them were adults [12–14]. It is challenging to explore the real cause of those abnormalities. Therefore, our current understanding of how ARS may affect the cornea is incomplete.

The characteristic histopathologic features of ARS are prominent, anteriorly displaced Schwalbe’s line and tissue strands connecting peripheral iris and corneal limbus. Moreover, it was observed that the abnormal Schwalbe’s line was composed of dense collagen and ground substance covered by a layer that resembled the Descemet membrane (DM) [13]. However, due to the small number of patients, there are lack histopathological observations focusing on the corneal opacity that primarily occurs in ARS.

PA is another group of ASD characterized by central corneal opacity with corresponding defects in the posterior stroma, DM and endothelium [15]. Some clinical features are common in both ARS and PA, such as the abnormal development of the trabecular meshwork and iris. However, compared to ARS patients, PA patients showed more frequent corneal abnormalities, thinner central corneal thickness with corresponding defects.

In this study, we analyzed 3 cases (6 eyes) of ARS with various degrees of corneal abnormalities. After keratoplasty, we performed a histopathological examination on corneal tissues and presented a new finding, which might improve our understanding of this disease.

## Materials and methods

Three patients (6 eyes) diagnosed with ARS complicating corneal opacity were retrospectively reviewed. All patients had undergone keratoplasty(penetrating keratoplasty, PK or Descemet stripping automated endothelial keratoplasty, DSAEK)unilaterally or bilaterally for irreversible endothelial decompensation at Peking University Third Hospital from May 2016 to January 2019. Besides, they had no surgical history prior to keratoplasty, which allowed us to observe the primary ocular appearance. The study was conducted following the revised Declaration of Helsinki. Ethics approval was obtained from the Institutional Review Board of Peking University Third Hospital. Informed consent was obtained from their guardians.

Patients were diagnosed with ARS if either of the following characteristics were observed in either eye [3, 16]: (1) Iris hypoplasia including loss of iris stroma, polycoria, corectopia and ectropion uveae; (2) An appearance of posterior embryotoxon under slit-lamp examination in combination with peripheral anterior synechia. Systemic features such as midface dysmorphism, dental abnormality, and redundant periumbilical skin helped diagnose ARS. To differentiate from PA, the patients exhibited the absence of the posterior corneal stroma, DM or endothelium were excluded.

The data gathered concerning demographics (age at operation, gender), family histories, systemic abnormalities, preoperative and intraoperative data, including best-corrected visual acuity (BCVA) (logMAR), intraocular pressure (IOP) (Goldmann tonometer), slit-lamp biomicroscopy findings, anterior segment optical coherence tomography (AS-OCT, Visante, Carl Zeiss Meditec, Dublin, CA, USA) photographs. Gene sequencing was recommended for all patients, yet none of them were tested partly for financial reasons.

### Histopathology of surgical specimens

Cornea tissue (full-thickness cornea or endothelium/DM) of these patients underwent histopathological examination after surgery. Histopathologic procedures, including hematoxylin and eosin (H&E) stain and periodic acid-Schiff (PAS) stain were performed to obtain the pathologic features of the cornea sections. All the sections were examined using light microscopy.

## Results

Four eyes of 2 males and 2 eyes of a female were included in our study. The mean age at operation was 7.0 ± 0.43 years (ranged 6.3–7.6 years). They were clinically diagnosed as ARS and showed characteristic systemic abnormalities, while none had a family history of ocular diseases. Two patients (Patient 1 and 3) were born with corneal opacity bilaterally. Except for the left eye of Patient 2, all eyes had developed irreversible endothelial decompensation. Three eyes (Patient 1 bilateral eyes, OU, Patient 2 right eye, OD) were treated by PK, while 2 eyes (Patient 3 OU) underwent DSAEK.

Among 5 eyes that developed irreversible endothelial decompensation, BCVA ranged from 0.9 to 0.2 logMAR before the occurrence of corneal edema and declined to a range of hand motions to 1.7 logMAR afterwards. Before the operation, all the eyes maintained normal IOP without medications.

### Clinical observation

**Fig. 1:**
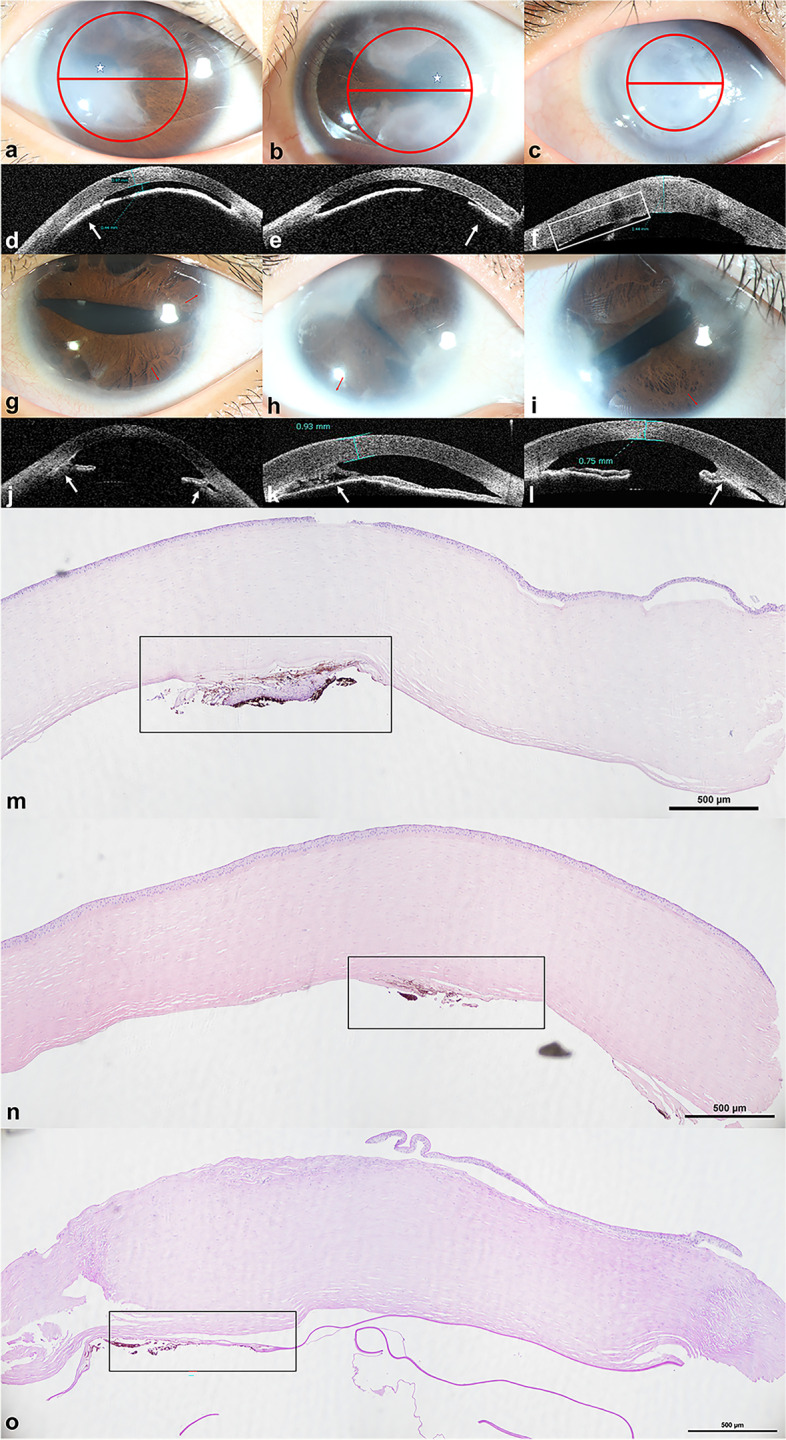
Clinical features of the patients (**a**, **d**, **m**, Patient 1 right eye, OD; **b**, **e**, **n**, Patient 1 left eye, OS; **c**, **f**, **o**, Patient 2 OD; **g**, **j**, Patient 2 OS; **h**, **k**, Patient 3 OD; **i**, **l**, Patient 3 OS) (**a**-**c**, **g**-**i**) Corneal opacities were observed under the slit lamp. In Patient 1, central and temporal opacities covering the pupil are shown, and the primary opacities are located in the inferior nasal and superior temporal quadrants in Patient 3. The right eye of Patient 2 exhibited severe corneal edema and opacity before keratoplasty, while the cornea was transparent in his left eye. Besides, iris abnormalities including corectopia (white mark), deformed pupil, polycoria, posterior embryotoxon (red arrow), iris atrophy and ectropion uvea could be seen. The red circle marked the excised cornea during PK, and the red line shows the section of histopathological examination. (**d**-**f**, **j**-**l**) Representative AS-OCT images. The images were taken in the transverse direction and showed shallow anterior chamber, thickened cornea and anterior synechia (white arrow). In both eyes of Patient 1 and Patient 3, anterior synechia predominantly occurred in the temporal quadrant, where corneal opacities existed. In the right eye of Patient 2, an obscure interface between the DM and the stroma could be observed (white square). (**m**-**o**) Overview images of both corneas of Patient 1 and the right cornea of Patient 2 under a light microscope. Edematous epithelium and stroma were shown. In Patient 1, the abnormal sections were in the temporal quadrant of both corneas, which was consistent with opaque areas (HE, original magnification × 40). In the right cornea of Patient 2, the Descemet membrane detachment (DMD) was observed with the peripheral interlayer splitting (PAS, original magnification × 40)

All eyes showed characteristic iris abnormalities under a slit lamp, including corectopia, polycoria, and stromal hypoplasia. Ectropion uveae was observed in the left eye of Patient 2. Both eyes of Patient 3 and the left eye of Patient 2 exhibited characteristic posterior embryotoxon, which was absent in Patient 1 (Fig. 1). In particular, patient 1 showed characteristic iris abnormalities (iris stromal hypoplasia and corectopia in both eyes) and typical systemic features (telecanthus, maxillary hypoplasia, microdontia et al.) of ARS. Therefore, a diagnosis of ARS was made clinically despite the absence of posterior embryotoxon.

Except for the left eye of Patient 2, corneas of all eyes presented with various degrees of edema. AS-OCT photographs of all eyes showed the peripheral anterior synechia, while in both eyes of 2 patients (Patient 1 and 3) the iris adhered to the quadrants where corneal opacities occurred (Fig. 1). All these eyes showed marked shallow anterior chamber and thickened cornea. Besides, an obscure interface could be seen on AS-OCT images of the right eye of Patient 2, indicating the mild detachment of DM.

Confocal imaging was performed on the left eye of Patient 2. We observed endothelial cells with a normal hexagonal pattern but indistinct boundary and no primary endothelial lesion. Meanwhile, the average cell density was 2303 cell/mm^2^. The average cell density continuously declined to 1309 cell/mm^2^ during a two-year follow-up period. Furthermore, the cornea remained transparent at the most recent follow-up, and the eye maintained normal IOP with regular use of topical ocular hypotensive medications.

### Histopathological findings

H&E and PAS stained sections of 5 eyes that underwent keratoplasty and were observed under a light microscope, showing roughly complete structures. The endothelium/DM specimen removed from both eyes of Patient 3 after DSAEK showed an uneven thickness of DM and an absence of endothelium.

**Fig. 2:**
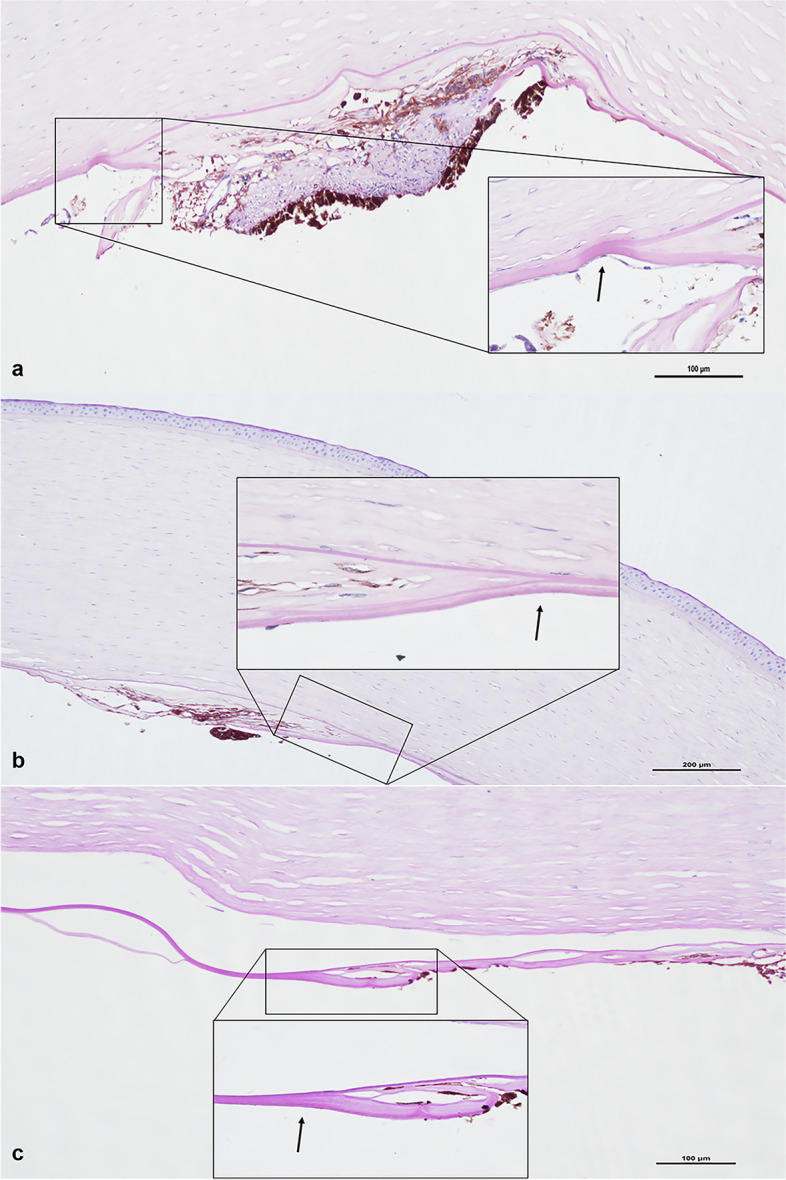
The novel histological finding of the excised cornea buttons after PK At high magnification, we observed the splitting of the DM (black arrow), where the hypoplastic iris and a uniform, homogeneous, lightly stained basement membrane-like structure were inserted. (**a**: Patient 1 OD, PAS, original magnification × 100; **b**: Patient 1 OS, PAS, original magnification × 100; **c**: Patient 2 OD, PAS, original magnification × 100)

In 3 eyes treated with PK (Patient 1 OU, Patient 2 OD), we observed the edematous epithelium, grossly normal or completely absent Bowman’s membrane, thickened stroma with disarranged fibers and increased stromal cells (Figs. 1 and 2).

Interestingly, we noted an anomalous structure in the 3 eyes treated by PK. The structure was observed in the area with corneal opacity and anterior synechia (Fig. 1). At high magnification, we found that the DMs of these 3 eyes were split and inserted by hypoplastic iris and a uniform, homogeneous, lightly stained basement membrane-like structure (Fig. 2). Besides, we observed the detachment of the DM in the right eye of Patient 2 (Fig. 1).

## Discussion

In this study, we retrospectively studied 3 cases of ARS together with corneal abnormalities. The patients in our study did not have a history of intraocular surgery. We hoped to observe the keratopathy and histopathological changes directly caused by ARS to deepen our understanding of this disease.

During embryonic development, the NCCs and mesoderm cells migrate to the area between the surface ectoderm and the optic cup, contributing to the corneal stroma and endothelium, the iris stroma and the trabecular meshwork [17]. The development process of the posterior cornea and the iris are closely related, and the abnormalities in these processes can lead to corneal opacity and anterior synechia. A previous study on congenital corneal opacity found that the severity of the corneal opacity was positively correlated with the amount of iridocorneal adhesions [18]. In our study, the opaque areas corresponded to the adherent areas, indicating a closer relationship between these two signs. It can be speculated that in some ARS patients, the adherence of iris may occur along with the corneal abnormalities during the embryonic period.

We made a novel histopathological finding which had never been reported before in ARS. In the eyes treated by PK, we observed the interlayer splitting of the DM with hypoplastic iris and a uniform, homogeneous, lightly stained basement membrane-like structure inserted in it. Besides, this feature happened to be in the area with corneal opacity and anterior synechia. Meanwhile, Ni et al. [19] had made an analogous histopathological finding in a child diagnosed with PA, which is also caused by abnormal migration and differentiation of ocular NC cells. They observed the “multiple-layer” structures at the peripheral part of the DM, with pigmented tissues inside the layers. The highly similar histopathological appearance may suggest the common underlying mechanism. Currently, it is commonly believed that three waves of cell migration produce anterior segment structures. Different waves of cells formed the corneal endothelium, the corneal stroma and the iris stroma, but these processes were under control of the same transcription factors and signaling pathways [17]. Accordingly, we speculated that the NCCs might migrate to the wrong location in these patients, leading to the mixture of hypoplasia iris and the DM. After that, the abnormal development of the NCCs caused the corneal opacities and anterior synechia at these sites. However, establishing the anterior ocular segment is a complicated process affected by multiple factors [20], and a complete understanding of this process remains elusive. Further studies are needed to illustrate the specific mechanism. Furthermore, we did not find the abnormal structure in the patient treated by DSAEK, where the procedures are more likely to ruin the primary structure of the posterior cornea and less likely to cover the abnormal area. Besides, this abnormal histopathologic feature may not appear in all ARS patients complicating corneal abnormalities.

Five eyes without the prior surgical history developed irreversible endothelial decompensation in our study. Endothelial decompensation had been found in several cases with ARS [8, 13, 21]. However, in these cases, the damage to endothelial membrane could be explained by surgery or long-standing glaucoma. Besides, corneal guttae and beaten metal appearance of corneal endothelium were observed in several cases of ARS [12–14, 21]; however, it is still unclear whether this endothelial appearance is related to endothelial decompensation. We examined the endothelial cells in the left eye of Patient 2, and recorded the subsequent changes over the following two years. The result showed the cells with a normal hexagonal pattern but obscure boundary. Besides, neither corneal guttae nor the beaten metal appearance of the corneal endothelium was observed. The continuous decline in the cell count might suggest the existing damage despite the transparent cornea. This slight but progressive endothelial damage may exist in other ARS patients, yet existing theories could not explain it. It is also hard to determine whether it primarily occurred or is the result of a progression of the disease. The tissue strands extending from the edge of the corneal endothelium to peripheral iris are a major characteristic of ARS [13]. Accordingly, we hypothesized that traction of these tissue strands might cause irreversible corneal decompensation in these eyes. These tissue strands moved with the pupil and gradually lost corneal endothelial cells near the adhesion area. The surrounding endothelial cells were filled in afterwards. We found no analogous assumption in previous studies, and more research is needed to verify our opinion. Additionally, the histopathologic examination found the DMD in the right eye of Patient 2, who exhibited diffuse edema. The AS-OCT images before operation showed a weak adhesion between DM and posterior corneal stroma. DMD had been reported after PK, especially in those with preexisting endothelial diseases [22]. Therefore, the operation procedures, pre-existing endothelial damage, and tissue strands’ long-standing traction might jointly lead to the DMD we observed.

Two major genes, forkhead box C1 (FOXC1) and pituitary homeobox 2 (PITX2), were proved to be associated with ARS [1]. It should be pointed out that mutations in the FOXC1 can cause a wide spectrum of clinical phenotypes. Segregation of both ARS and PA was observed in the same family or the same patient with a single point mutation of FOXC1 [4, 15, 23]. Well aware of the importance of the genetic testing for ARS patients, we recommended it in all patients clinically diagnosed as ARS. Unfortunately, none of the patients included in our study had genetic testing partly for financial reasons. We expect that future research would help to refine our findings.

## Conclusion

In conclusion, 6 eyes with ARS were specifically described in our study. We focused on congenital corneal opacity and irreversible endothelial decompensation in these eyes and explored the underlying mechanism through histopathologic examination. Consequently, we made a novel histopathologic finding and found progressive endothelial damage in 1 patient. To summarize our finding, corneal abnormalities in ARS patients can be primary or secondary. We speculated that the long-lasting traction from the tissue strands connecting the cornea and the iris may cause the loss of endothelial cells, playing a fundamental role on the corneal decompensation observed in ARS patients. Our results may enhance the understanding of ARS and improve the prognosis of the patients with ARS complicating corneal abnormalities.

## Data Availability

The dataset used and/or analyzed during the current study are available from the corresponding author on reasonable request.

## Abbreviations

ARS: Axenfeld-Rieger syndrome
ASD: Anterior segment dysgenesis
NCCs: Neural crest cells
CCO: Congenital corneal opacity
PA: Peters anomaly
DM: Descemet membrane
PK: Penetrating keratoplasty
DSAEK: Descemet stripping automated endothelial keratoplasty
BCVA: Best-corrected visual acuity
IOP: Intraocular pressure
AS-OCT: Anterior segment optical coherence tomography
H&E: Hematoxylin and eosin
PAS: Periodic acid-Schiff
OU: Bilateral eyes
OD: Right eye
OS: Left eye
DMD: Descemet membrane detachment.
